# KCNN4 may weaken anti-tumor immune response via raising Tregs and diminishing resting mast cells in clear cell renal cell carcinoma

**DOI:** 10.1186/s12935-022-02626-7

**Published:** 2022-06-10

**Authors:** Yankang Cui, Tianyi Shen, Feng Xu, Jing Zhang, Yuhao Wang, Jiajin Wu, Hengtao Bu, Dian Fu, Bo Fang, Huichen Lv, Suchun Wang, Changjie Shi, Bianjiang Liu, Haowei He, Hao Tang, Jingping Ge

**Affiliations:** 1 Department of Urology, Clinical School of Medical College, Jinling Hospital, Nanjing University, Nanjing, China; 2 School of Chemistry and Chemical Engineering, Jiangsu Province Hi-Tech Key Laboratory for Biomedical Research, Southeast University, Nanjing, China; 3 Department of Urology, The First Affiliated Hospital of Nanjing Medical University, Nanjing, China

**Keywords:** Renal cancer, ceRNA, Immune cells, KCNN4, Prognosis

## Abstract

**Background:**

Studies over the past decade have shown that competitive endogenous RNA (ceRNA) plays an essential role in the tumorigenesis and progression of clear cell renal cell carcinoma (ccRCC). Meanwhile, immune checkpoint blocker is gradually moving towards the first-line treatment of ccRCC. Hence, it’s urgent to develop a new prediction model for the efficiency of immunotherapy. At present, there is no study to reveal the effect of ceRNA network on the efficiency of immunotherapy for ccRCC.

**Methods:**

To systematically analyze the effect of ceRNA hub genes in ccRCCon immune response, we constructed prognosis models based on ceRNAs and immune cells, respectively. We constructed ceRNA network using hypergeometric distribution test and correlation analysis with R script based on The Cancer Genome Atlas (TCGA) database. We then applied the Cibersort algorithm to simulate the infiltration overview of immune cells in kidney renal clear carcinoma (KIRC) samples. Prognosis-related immune cells were screened and a predictive model of these cells was constructed. Prognosis-related immune cells and ceRNA hub genes were performed with co-expression analysis. Finally, qRT-PCR and immunofluorescence assays were performed to validate the results.

**Results:**

The construction of ceRNA related prognosis model contained 8 hub genes, including RELT, MYO9B, KCNN4, SIX1, OTOGL, MALAT1, hsa-miR-130b-3p, and hsa-miR-21-5p. The area under the receiver operating characteristic curve (AUC) was 0.77 at 5 years. For the construction of immune cells prognosis model, 3 immune cells (T cells regulatory, Macrophages, Mast cells resting) were adopted, and the AUC was 0.65 at 5 years. We then merged the two models by correlation analysis and co-expression analysis. Finally, we found that KCNN4 positively correlates with T cells regulatory (Tregs) and negatively correlates with mast cells resting significantly. Furthermore, higher expression of KCNN4 may lead to a higher potential for immune evasion and lower efficiency for immune checkpoint inhibitors (ICIs).

**Conclusions:**

Generally, this is the first study to assess the prognostic value of immune related ceRNA hub genes in ccRCC, and KCNN4 was finally demonstrated to be a key regulatory factor with strong correlation with Tregs and mast cells resting.

**Supplementary Information:**

The online version contains supplementary material available at 10.1186/s12935-022-02626-7.

## Background

Renal cancer is one of the ten most common cancers worldwide and accompanies with about 85,680 estimated new cases in the United States, in 2020 [1]. Among which, clear cell renal cell carcinoma (ccRCC) is the most commonly cancer subtype and accounts for most of the cancer-related deaths [2]. Advanced renal cancer gives rise to considerable morbidity and mortality. However, in the past decade, advances in treatment have substantially ameliorated the overall survival (OS) of patients with this disease. Particularly, immune checkpoint inhibition has proven to be an important and effective new strategy in the management of patients with advanced renal cancer.

RCC has a poor response to conventional chemotherapeutics. Although treatments that target vascular endothelial growth factor (VEGF) and rapamycin (mTOR) have increased therapeutic options, practically all patients eventually acquire resistance to these antiangiogenic or molecularly targeted therapies [3]. Unlike chemotherapeutics, immunotherapy is expected to be a reliable choice for patients with advanced RCC. In 2019, the Food and Drug Administration (FDA) approved combinatorial approaches which incorporating PD1 blockade and anti-angiogenic therapy as the first-line treatment for patients with advanced RCC [4]. However, current systemic immune treatments were limited by low response rates and toxic adverse effects [5]. Aside from immune checkpoint inhibition, the tumor microenvironment has been highlighted to influence immunotherapeutic response and prognosis through various signaling pathways [6]. Hence, more studies on identifying high-performance biomarkers which reflect tumor microenvironment and predict patients’ sensitivity to immunotherapy may help us develop novel therapies for ccRCC treatments.

Soon after the novel ceRNA notion was proposed [7], increasing bioinformatics data or experimental results have reported that most protein-coding genes and cancer-related lncRNAs in the human genome densely contain miRNA recognition elements (MREs), which validates the existence of lncRNA‒miRNA‒mRNA logic axis in cancers, including RCC. lncARSR was reported to act as a ceRNA for miR-34 and miR-449 to promote AXL and c-MET expression, and so contribute to sunitinib resistance in RCC [8]. LINC00973 could function as ceRNA to regulate cell surface abundance of Siglec‐15 through sponging miR‐7109. LINC00973‐miR‐7109‐Siglec‐15 axis was involved in immune activations in ccRCC [9]. Additionally, in recent years, some studies used bioinformatic methods to construct ceRNA networks which displayed satisfactory prognostic value for ccRCC [10, 11]. All these findings drove us to explore the connection between ceRNA network and tumor immune microenvironment.

In this study, we first constructed ceRNA networks and screened prognosis-related hub genes (HGs), and used these HGs to construct a predictive model. We then applied the cibersort algorithm to simulate the infiltration overview of immune cells in ccRCC samples. Prognosis-related immune cells were screened and a predictive model of these cells was constructed. We merged the above two models and searched for the HGs that were highly correlated with immune cells. Finally, KCNN4 was found to recruit Tregs and restraint mast cells resting. The followed experiments verified this. Briefly, KCNN4 may weaken anti-tumor immune response via raising Tregs and diminishing resting mast cells in ccRCC. The miR-16-5P was predicted to bind to the 3’-UTR of KCNN4 and so release the immunosuppression.

## Methods

### Workflow

**Fig. 1:**
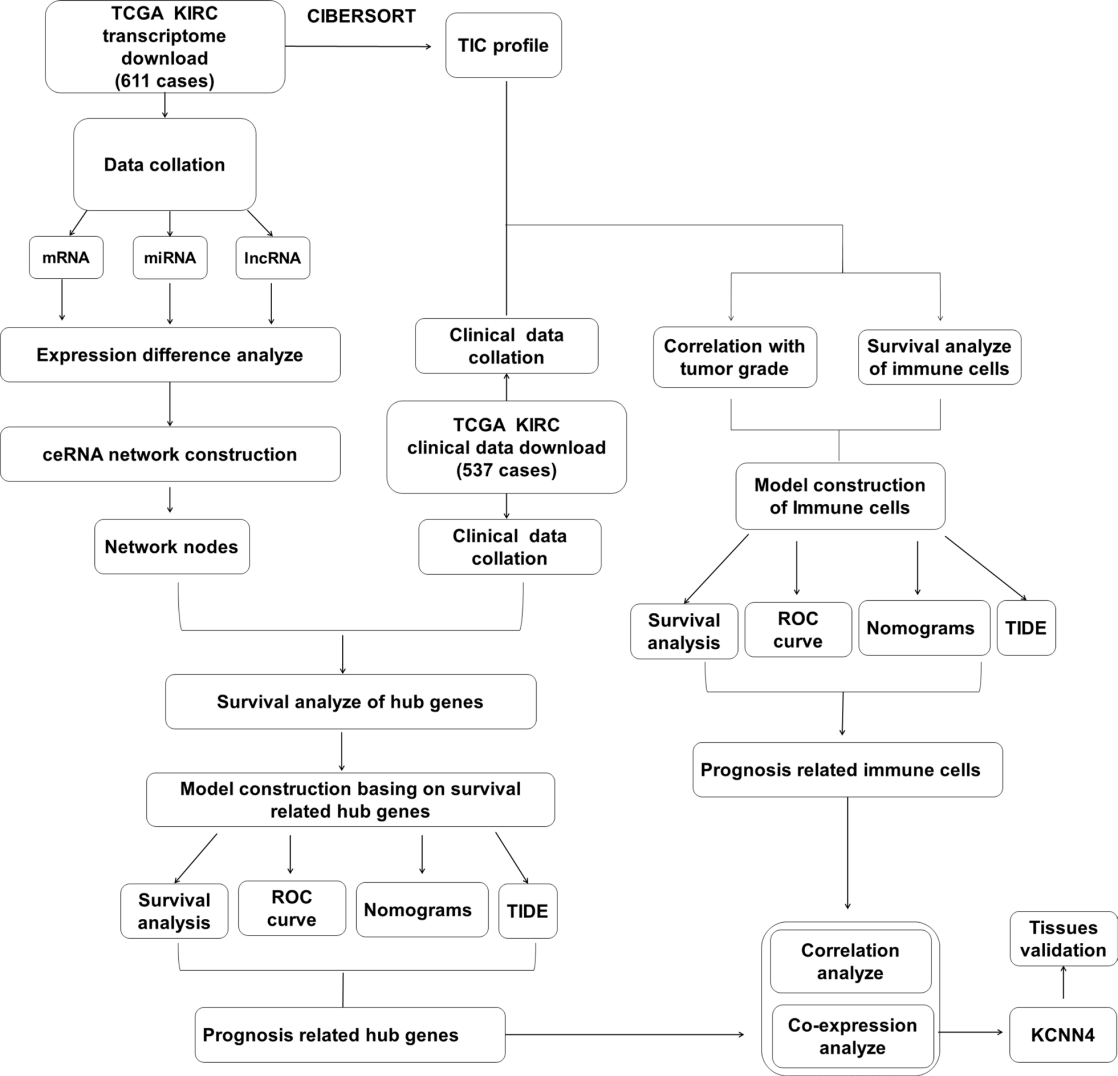
Main workflow for the study. (TIC: tumor-infiltrating immune cells)

The steps we used in this study to investigate the ceRNA network hub genes’ immune signature were displayed in Fig. 1.

### Data collection and pre-processing

Transcriptome profiling data (Count) of KIRC was downloaded from TCGA (https://portal.gdc.cancer.gov/) and divided into mRNA, miRNA and lncRNA with R algorithms using human.gtf. The corresponding clinical information of these cases was also collected.

### ceRNA network construction

Given the mRNA, miRNA and lncRNA expression matrix, differentially expressed genes were screened with ‘DEseq2’ package and extracted to build ceRNA network. The fold-change > 2 and P value < 0.05 was considered as significant. For preliminary forecasting the relationship of lncRNA-miRNA and miRNA-mRNA, we used ‘starbase’ database and got an initial network. Then we conducted hypergeometric distribution test and correlation analysis to filter the network, and the p value cutoff was 0.001. Finally, the network was drawn with Cytoscape software (v3.8.2).

### The overall survival analyses of the ceRNA network hub genes

Given the expression matrix of ceRNA network hub genes and clinical data, we next analyzed the survival impact of these genes on KIRC patients. A total of 41 hub genes and 611 cases were included in the study cohort. ‘Survival’ package was used and p value < 0.05 was considered significant.

### Prognosis model of hub genes construction

To screen the prognosis related hub genes from above 18 survival related genes and construct the prognosis model, we conducted univariate COX proportional hazards regression analysis, lasso regression and multivariate COX regression analysis. The p value filter for univariate COX regression was 0.05 and significant genes were further enrolled to conduct lasso regression. The Akaike information criterion (AIC) values were used to optimize the multivariate COX model and the hub genes with the lowest AIC were retained in the final signature. The risk score formula was as followed: Risk score= ∑Gene_(exp)_×Gene_(coef)_ (Gene_(exp)_ indicates the expression of every single hub gene and the Gene_(coef)_ was calculated using a multivariable COX proportional hazards model). Afterward, patients were divided into high or low-risk group basing on the median risk score, the survival and receiver operating characteristic (ROC) analyses were subsequently performed to assess the model’s prognostic value for KIRC patients. The ROC analysis was performed with ‘timeROC’ package. A nomogram summarizing the multivariate COX model was developed with the ‘rms’ package in R 4.0.3. The correlation between gene risk score and tumor grade was performed with “limma” and “ggpubr” package. Finally, the TIDE tool (http://tide.dfci.harvard.edu/) from the Harvard University was used to assess the clinical efficiency of immune checkpoint inhibition therapy, where higher TIDE predictive scores correlated with poor therapeutic effect and worse prognosis.

### Prognosis model of immune cells construction

CIBERSORT was used to assess the infiltration level of 22 tumor immune cells in KIRC patients, and the samples whose p-value < 0.05 were incorporated to display the distribution difference of immune cells between tumor and normal samples by heatmap and violin plots. Besides, the correlation between immune cells in KIRC samples was analyzed with the ‘corplot’ package. We then performed survival analyses to get the relationship between immune cells infiltration level and overall survival time. Afterward, clinical prognostic factor-tumor grade was also included as a measure to further assess the prognostic value of above survival-related immune cells. For model construction, as well as above hub genes, univariate COX proportional hazards regression analysis, lasso regression and multivariate COX regression analyses were carried out in succession. The immune cells with the lowest AIC were retained in the final signature. After constructing the model, we assessed this model via OS, ROC, nomogram, clinical correlation, and TIDE analyses same as the hub genes model based on risk score with R algorithm.

### The co-expression analysis of prognosis related immune cells and ceRNA network hub genes

Given the prognosis related ceRNA network hub genes and immune cells, we next conducted the co-expression analysis between them to find the potential hub genes related to immune cells regulation with R algorithms. The Pearson correlation coefficient (R) was calculated to establish whether there was a correlation between hub genes and immune cells. |R|> 0.3 and p value < 0.001 were considered as significant. Timer database (http://timer.cistrome.org/) was used to validate the co-expression relationships between hub genes and immune cells markers in KIRC samples. Ualcan database (http://ualcan.path.uab.edu/) was used to analyze the expression level of KCNN4 in different tumor grades.

### Gene set enrichment analysis (GSEA)

611 KIRC samples in TCGA were divided into two groups (high and low KCNN4 expression) based on the median expression. We conducted GSEA between the two groups to identify the significantly altered Gene Ontology (GO) pathways by using GSEA software (v4.0.3). C5.all.v6.1.symbols.gmt was used as the gene set. The p and FDR q values were obtained from 1,000 permutations and P < 0.05 was considered statistically significant.

### Tumor microenvironment analysis

We used ESTIMATE algorithm (Estimation of Stromal and Immune cells in Malignant Tumor tissues using Expression data) algorithm to measure stromal and immune scores in KIRC with data from TCGA database.

### Sample collection, RNA extraction and quantitative RT-PCR

The ccRCC normal and tumor tissues were acquired from patients who were diagnosed with ccRCC and underwent surgery at The First Affiliated Hospital of Nanjing Medical University between 2015 and 2020. Patients who were diagnosed with ccRCC by pathology were included, and those who had any medical history of other neoplasms was excluded. Finally, a total of 40 pairs of tissues were included in our cohort. Samples for RNA extraction were freshly frozen in liquid nitrogen and stored at − 80 ℃. Samples for immunofluorescence analysis were formalin fixed. The study design and protocol were approved by the ethics committee of The First Affiliated Hospital of Nanjing Medical University. All patients included in this study provided informed consent. RNA-Quick purification kit (YISHAN, ShangHai, China) was used to extract and purify the samples’ total RNA according to the manufacturer’s protocols. Briefly, lysis buffer was used to split the tissues. Wash buffer was used to remove excess impurities, and elution buffer was used to dissolve the total RNA. The total RNA was reverse transcribed into complementary DNA (cDNA) using HiScript.

II (Vazyme, Shanghai, China). qRT-PCR was performed using SYBR Green I (Vazyme, Shanghai, China) on ABI 7900 system (Applied Biosystems, Carlsbad, CA, USA) and the primers for KCNN4 were as follows: forward (F), 5′-CTGCTGCGTCTCTACCTGG-3′; reverse (R), 5′-AGGGTGCGTGTTCATGTAAAG-3′.

### Immunofluorescence

The tissues which fixed in formalin were made into paraffin embedded tissue blocks and sliced. After washed with PBS for 3 times, the slices were blocked with 10% goat serum for 45 min. Then, the slices were incubated with indicated primary (Proteintech; 23,271–1-AP) and secondary antibodies at 4 °C overnight or at room temperature for 2 h, respectively. The nucleus was stained with DAPI and signals were observed using a fluorescence microscope (Nikon).

### Statistical analysis

Almost all analyses were performed with R software (4.0.0) or Perl (5.32.0) script. Usually, Perl script was used to process data, such as gene id transformation, data merging etc. The Student’s t-test was used to compare the differences between two or three groups. P value < 0.05 was considered statistically significant unless noted otherwise.

## Results

### The predictive model we constructed based on ceRNA hub genes has significant prognostic value for KIRC patients

**Fig. 2:**
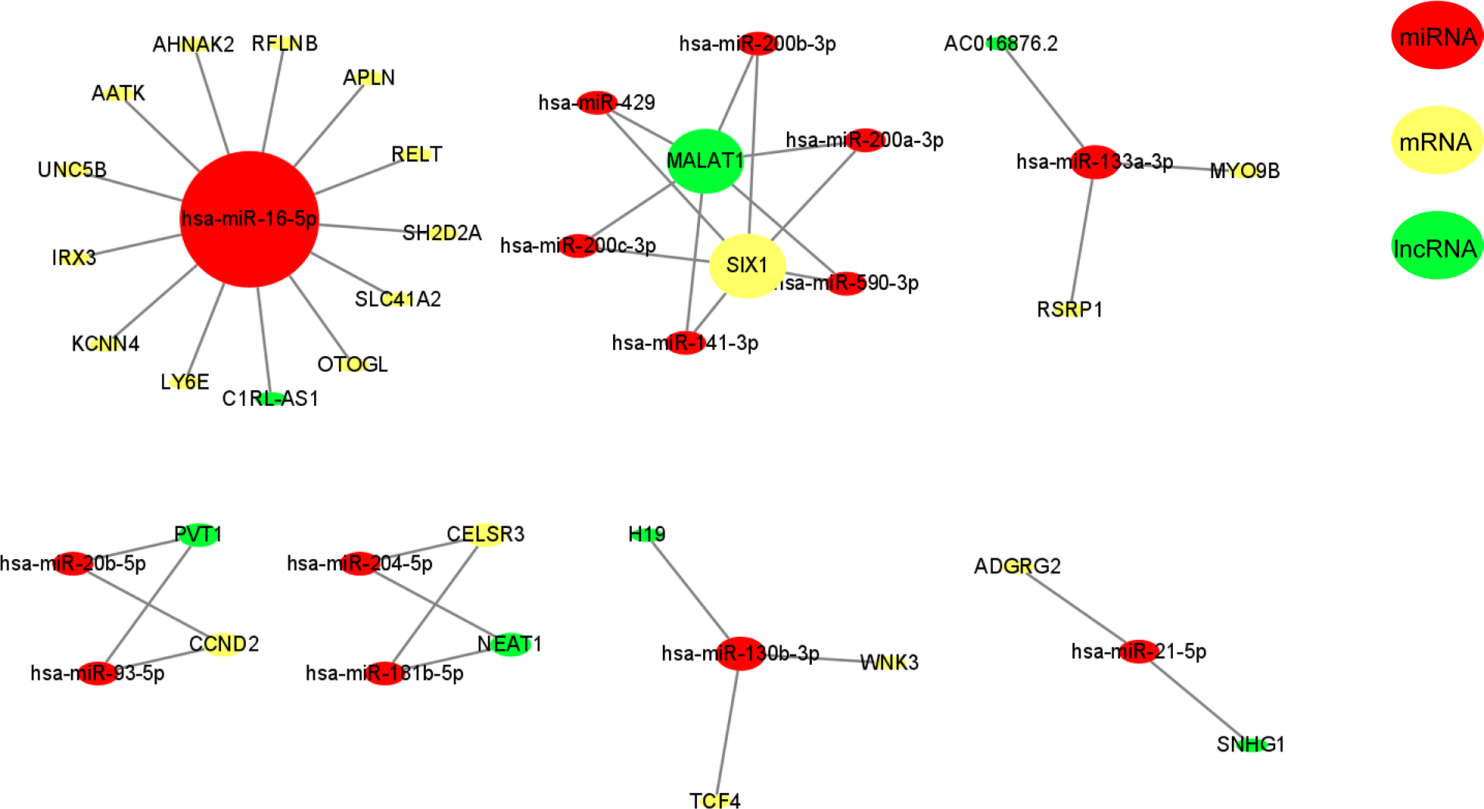
Networks of ceRNAs predicted by hypergeometric distribution test and correlation analysis. The red ellipse indicates miRNA. The yellow ellipse indicates mRNA. The green ellipse indicates lncRNA. ceRNA: competitive endogenous RNA

Different expressing genes in KIRC tumors and normal tissues were analyzed with R algorithm and showed in Additional file 1: Figure S1. After hypergeometric distribution test and correlation analysis, a total of 20 mRNA, 14 miRNA, and 7 lncRNA were filtered out to construct the ceRNA network (Fig. 2). We then performed survival analyses with these HGs to get the survival related ones. As showed in Additional file 2: Figure S2, 18 HGs were found to be significant related to ccRCC patients’ survival probability. Univariate COX proportional hazards regression analysis was conducted with these HGs for the initial screening of prognosis related HGs (Table 1).

**Table 1:** Univariate COX proportional hazards regression analysis of HGs

Id	HR	HR.95L	HR.95H	p value
CELSR3	1.30825	1.16335	1.47120	7.25E-06
SH2D2A	1.25329	1.12161	1.40042	6.71E-05
RELT	1.96307	1.57015	2.45431	3.23E-09
MYO9B	1.43954	1.05476	1.9647	0.021679
KCNN4	1.52858	1.34385	1.73870	1.07E-10
UNC5B	0.84574	0.73655	0.97112	0.017532
RSRP1	1.30938	1.12468	1.52442	0.000512
SIX1	1.20422	1.05746	1.37136	0.005069
OTOGL	0.77371	0.69679	0.85912	1.57E-06
APLN	0.86073	0.76520	0.96818	0.012468
RFLNB	0.76791	0.66271	0.88982	0.000444
TCF4	0.75146	0.65271	0.86514	7.03E-05
NEAT1	1.16812	1.04117	1.31055	0.008114
PVT1	1.59150	1.31114	1.93181	2.60E-06
MALAT1	1.25327	1.10992	1.41513	0.00027
SNHG1	1.43115	1.17195	1.74767	0.000437
AC016876.2	1.33913	1.10733	1.61945	0.002601
hsa-miR-130b-3p	1.80859	1.52765	2.141209	6.01E-12
hsa-miR-200b-3p	0.875982	0.768244	0.998828	0.047988
hsa-miR-204-5p	0.897177	0.849	0.948088	0.000117
hsa-miR-21-5p	1.75617	1.433561	2.151378	5.40E-08
hsa-miR-590-3p	1.473438	1.17421	1.84892	0.000818

**Fig. 3:**
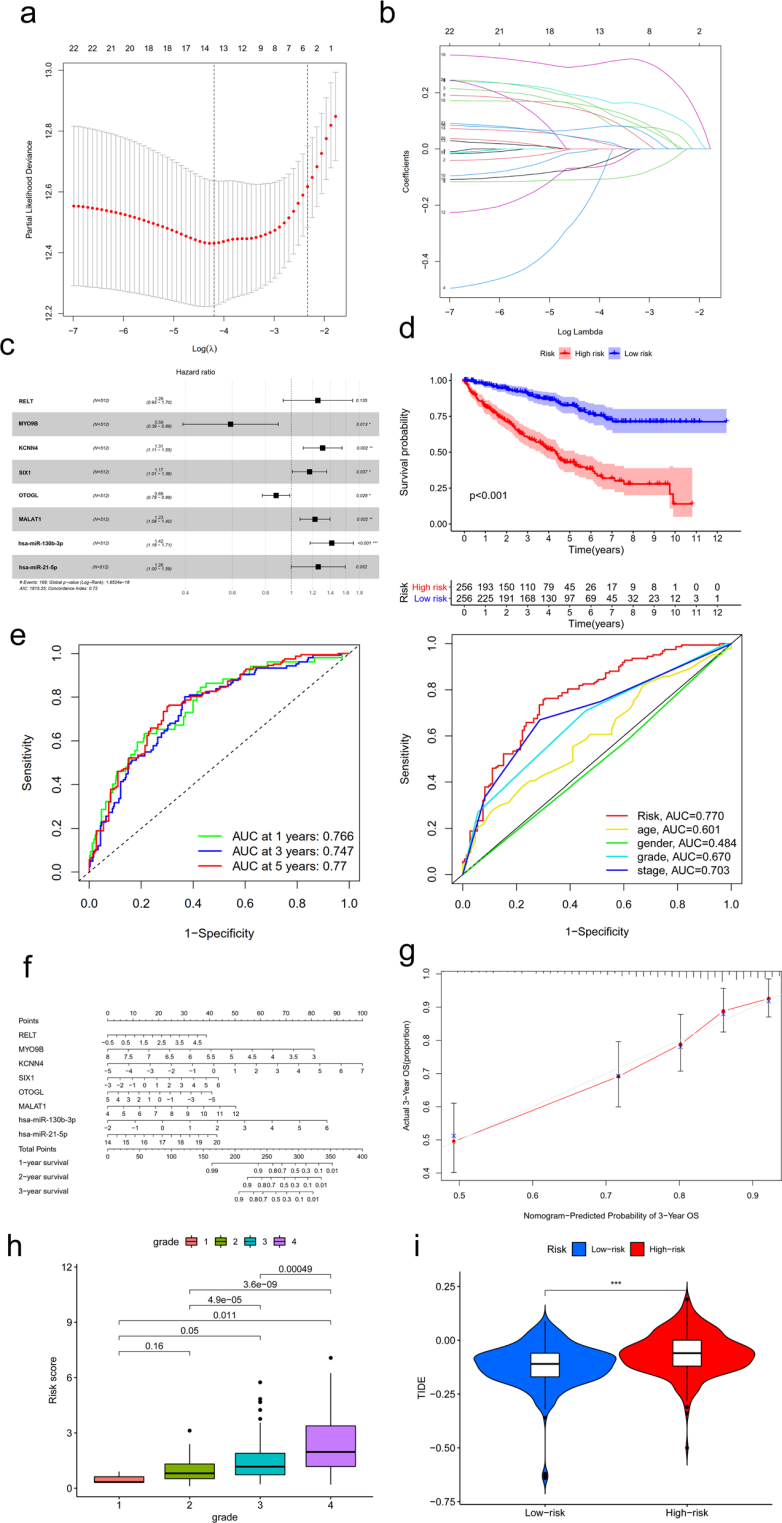
The construction of ceRNA prognosis model. **a, b** The results of the LASSO logistic regression. **c** The results of the multivariate Cox regression. Kaplan–Meier survival curve (**d**), model diagnosis process (**e**), and nomogram (**f**) of the prognosis model based on ceRNA prognosis model. **g** The calibration curve of the 3-year overall survival (OS) in ccRCC. (h) The risk score of hub genes model in KIRC based on tumor grade. (i) The relationship between risk score and TIDE score. LASSO: least absolute shrinkage and selection operator. SE = standard error

To avoid the influence of confounding factors, we performed a LASSO regression analysis to re-assess the HGs, 14 of them with nonzero coefficients (Fig. 3a, b). After evaluated with AIC value, we retained 8 HGs as potential prognosis related HGs of the prediction model. These HGs included RELT, MYO9B, KCNN4, SIX1, OTOGL, MALAT1, hsa-miR-130b-3p, hsa-miR-21-5p. Subsequently, we performed a multivariable COX regression analysis, constructed a predictive model. Except for RELT, hsa-miR-21-5p, the other 6 HGs showed a significant relationship with prognosis (Fig. 3c). The 8 HGs were then recruited to build the predictive model. In this model, we divided patients into high-risk group, low-risk group basing on their risk scores. The survival analysis revealed that high-risk patients have shorter survival time compared to low-risk patients (Fig. 3d, p < 0.001). ROC curve for the predictive model were showed in Fig. 3e. The AUC were 0.766, 0.747, 0.77 at 1, 3, 5 years, respectively. Compared to clinicopathological characteristics including tumor grade stage, the AUC for our risk score showed a higher value. Nomograms of the multivariable predictive model were prepared to graphically illustrate the relative impact of each HG to predict 1-, 2-, 3-year survival (Fig. 3f). The calibration curve of the 3-year OS suggested great fitness between actual 3-year OS, nomogram-predicted probability of 3-year OS. Besides, high risk score predicted high tumor grade (Fig. 3h), high TIDE score (Fig. 3i), which means poor immune checkpoint inhibition therapy effect, worse prognosis

### The predictive model we constructed based on immune cells has significant prognostic value for ccRCC patients

We estimated the abundance of various types of immune cell with cibersort for TCGA cohort, and the sum of the immune cell composition in each of the 22 samples was 100% (Additional file 3: Figure S3a). The difference of immune cells’ infiltration between tumor and normal samples was shown as a heatmap in Additional file 3: Figure S3b and a violin plot in Additional file 3: Figure S3c. The Additional file 3: Figure S3d displayed the correlation between these 22 subtypes of immune cells in ccRCC samples. Red indicates positive correlation, and blue indicates negative correlation. The correlation coefficients were also provided.

**Fig. 4:**
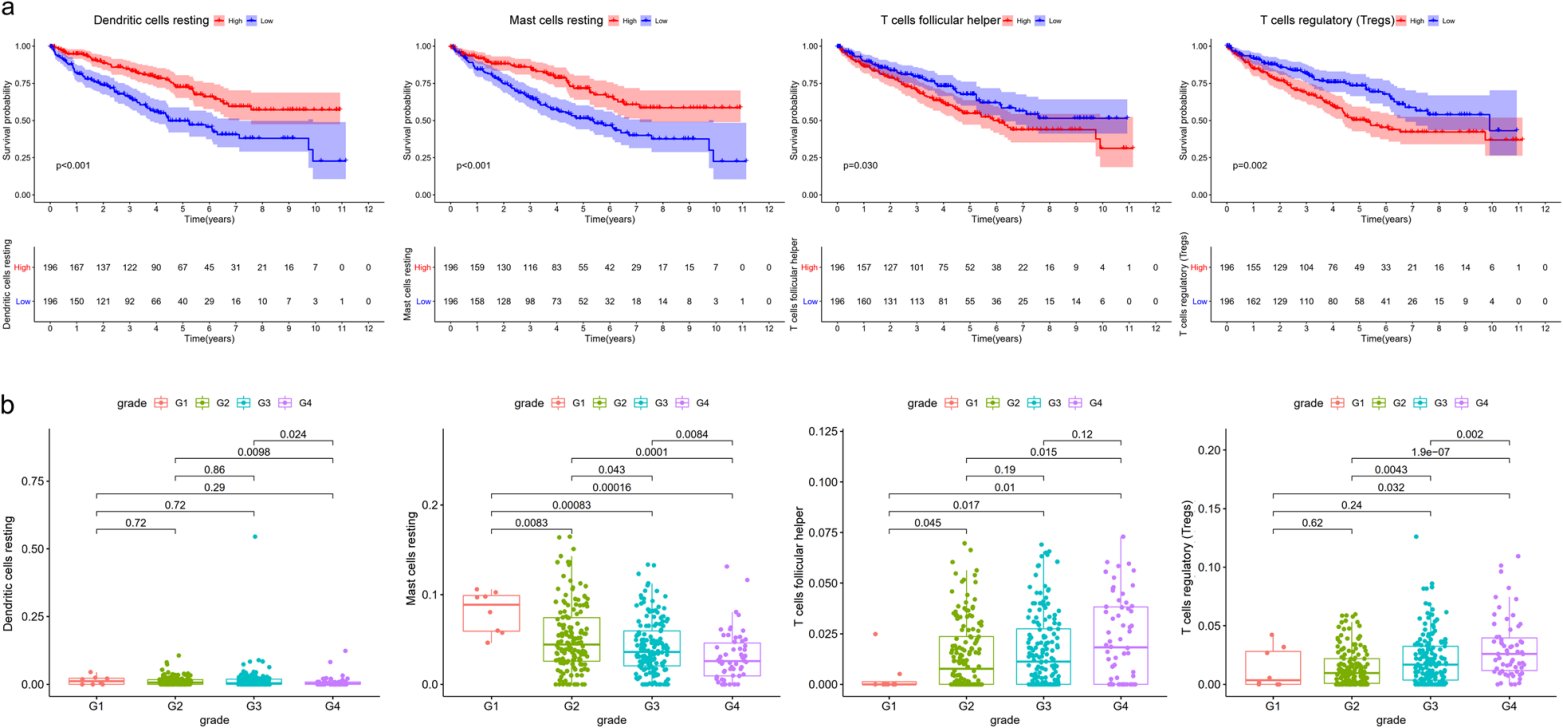
The assessment and screening of immune cells’ prognostic value for ccRCC patients based on OS (**a**) and tumor grade (**b**)

Given the overview of immune cells’ infiltration in ccRCC samples, we then evaluated the prognostic value of these immune cells in combination with the clinical information of the samples which also obtained from TCGA database. The survival analysis revealed a better survival probability in patients with higher infiltration of dendritic cells resting and mast cells resting (Fig. 4a, p < 0.001). On the contrary, higher infiltration of T cells follicular helper and Tregs will bring lower survival rate (T cells follicular helper: p = 0.03; Tregs: p = 0.002). Figure 4b described the 4 immune cells’ infiltration levels between different degrees of tumor grade. For dendritic cells resting, grade 4 (G4) samples showed lower infiltration than that in grade 2 (G2, p = 0.0098) and grade 3 (G3, p = 0.024) samples. Besides, with the increase of samples’ grade, the infiltration degree of mast cell resting gradually decreased. Briefly, for T cells follicular helper and Tregs, a higher infiltration level was represented in higher tumor grade.

**Fig. 5:**
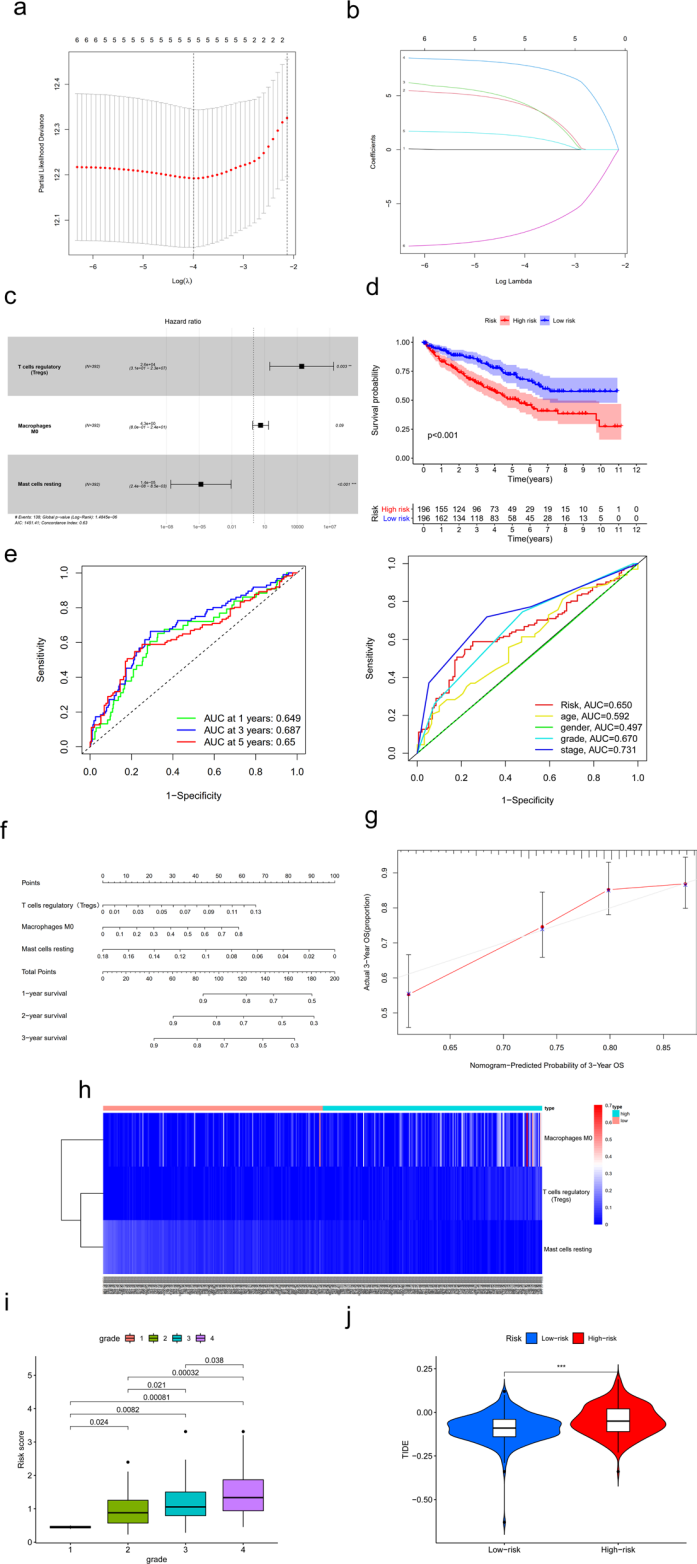
The construction of immune cells prognosis model. **a**, **b** The results of the LASSO logistic regression. **c** The results of the multivariate Cox regression. Kaplan–Meier survival curve (**d**), model diagnosis process (**e**), and nomogram (**f**) of the prognosis model based on immune cells prognosis model. **g** The calibration curve of the 3-year OS in ccRCC. **h** Distribution of prognosis related immune cells in high-risk and low-risk groups. **i** The risk score of immune cells model in KIRC based on tumor grade. **j** The relationship between risk score and TIDE score

Same as the construction of HGs’ predictive model, univariate COX proportional hazards regression analysis was used as the initial screening (Table 2). LASSO regression analysis indicated 5 of them with nonzero coefficients (Fig. 5a, b). Combined with AIC value’s assessment, 3 immune cells were retained for next multivariable COX regression analysis and the construction of predictive model. As showed in Fig. 5c, Tregs and mast cells resting had significant prognostic value for ccRCC. The predictive model we built based on the 3 immune cells displayed a good prognostic value according to the survival analysis and ROC curve analysis. High risk group had shorter survival time (Fig. 5d,  p< 0.001). AUC were 0.649, 0.687, and 0.65 at 1, 3, and 5 years, respectively (Fig. 5e). Nomograms of the multivariable predictive model were provided in Fig. 5f to graphically state the relative impact of each immune cells to predict 1-, 2- and 3-year survival. The calibration curve was also plotted to describe the fitness between the actual 3-year OS and the nomogram-predicted probability of 3-year OS. Furthermore, the macrophages M0 cells and Tregs showed a higher infiltration in high-risk group (Fig. 5h). The mast cells resting showed a higher infiltration in low-risk group. Lastly, same as hub genes predictive model, high risk score of immune cells predictive model tended to higher tumor grade (Fig. 5i) and worse efficiency of immune checkpoint inhibitors (ICIs, Fig. 5j).

**Table 2:** Univariate COX proportional hazards regression analysis of immune cells

Id	HR	HR.95L	HR.95H	p value
T cells CD4 memory resting	0.138211	0.020692	0.923158	0.041103
T cells CD4 memory activated	127607.1	45.60157	3.57E + 08	0.003693
T cells follicular helper	579694.1	124.2054	2.71E + 09	0.00208
T cells regulatory (Tregs)	670390.8	1105.508	4.07E + 08	4.07E-05
Macrophages M0	5.041978	1.020671	24.90669	0.047137
Mast cells resting	2.22E-06	4.40E-09	0.001122	4.16E-05

### KCNN4 may recruit Tregs and diminish resting mast cells to weaken anti-tumor immune response

**Fig. 6:**
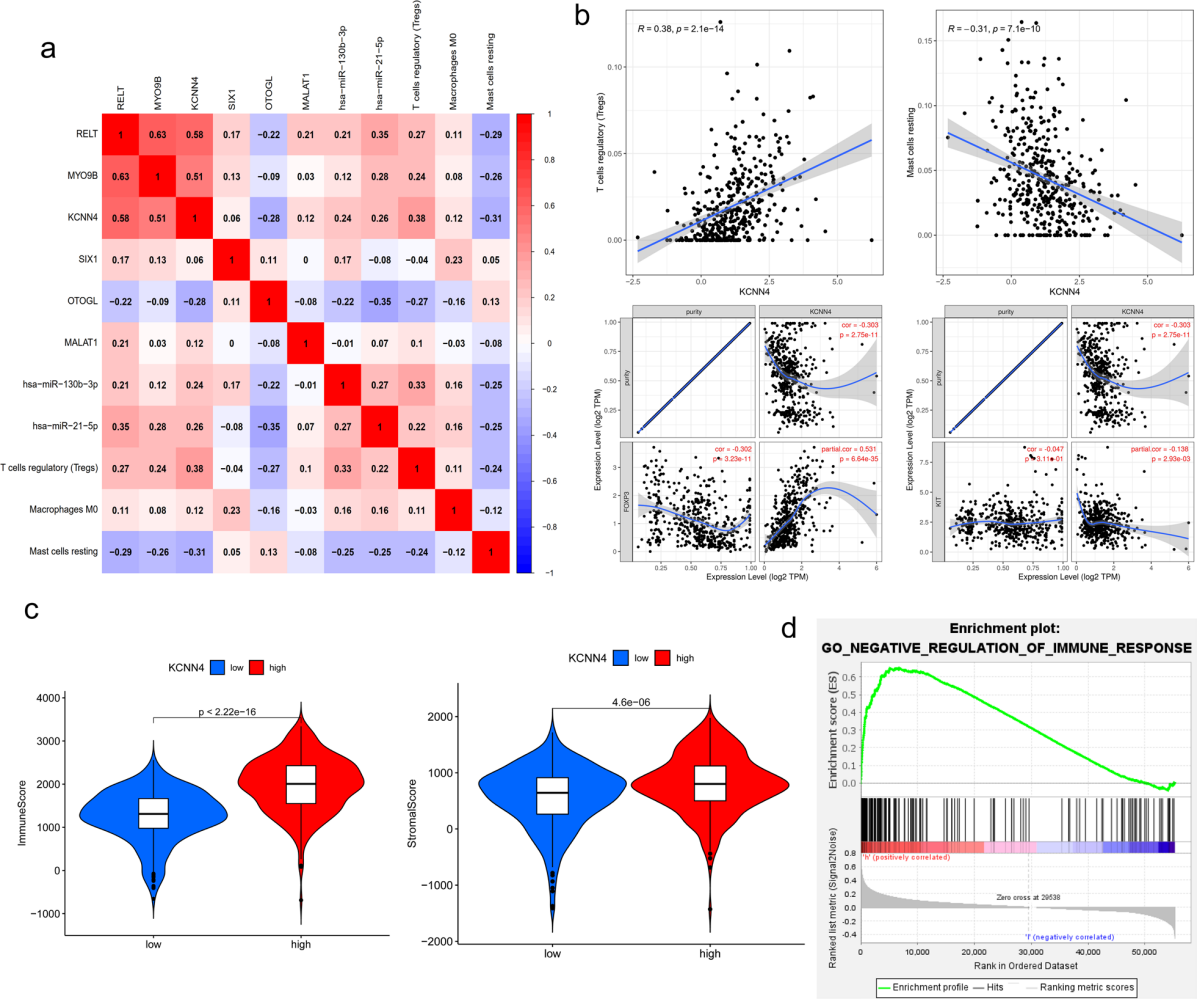
The merge results of the above two prognosis models. **a** The correlation analysis (**a**) and co-expression analysis (**b**) of prognosis-related ceRNA hub genes and immune cells. Further verification of the co-expression relationship between KCNN4 and the marker of Tregs (KCNN4), mast cells resting (KIT). **c** The correlation analysis between TME and KCNN4 expression. **d** The GSEA analysis of KCNN4 in ccRCC samples

Given the above 2 predictive models, we then merged them to find the immune related HGs via correlation analysis and co-expression analysis. Noteworthily, KCNN4 displayed a positive correlation with Tregs (Fig. 6a, b; R = 0.38, p = 2.1e-14) and a negative correlation with mast cells resting (R = -0.31, p = 7.1e-10). Subsequently, we used Timer database [12] to calculate the correlation between the expression of KCNN4 and the marker of Tregs (FOXP3), resting mast cells (KIT). The correlation was adjusted by tumor purity. The results are consistent with our analysis. KCNN4 correlated positively with FOXP3 (R = 0.56, p = 2.3e-45) and negatively with KIT (R = −0.16, p = 0.00016). Tumor microenvironment (TME) acts as a complex ecosystem, which could support tumor growth as well as metastasis while attenuating immunosurveillance. TME is a cellular environment consisting of tumor cells and other non-malignant cells, including surrounding immune cells, stromal cells, etc. We used the ESTIMATE algorithm to assess the impact of KCNN4 on TME. We found that the immunescore and stromalscore were elevated in samples which higher expressed KCNN4.

GSEA analysis further supported these findings as it showed a negative regulation of immune response enriched in KCNN4 positively correlated site. Hence, higher expression of KCNN4 may inhibit immune response.

**Fig. 7:**
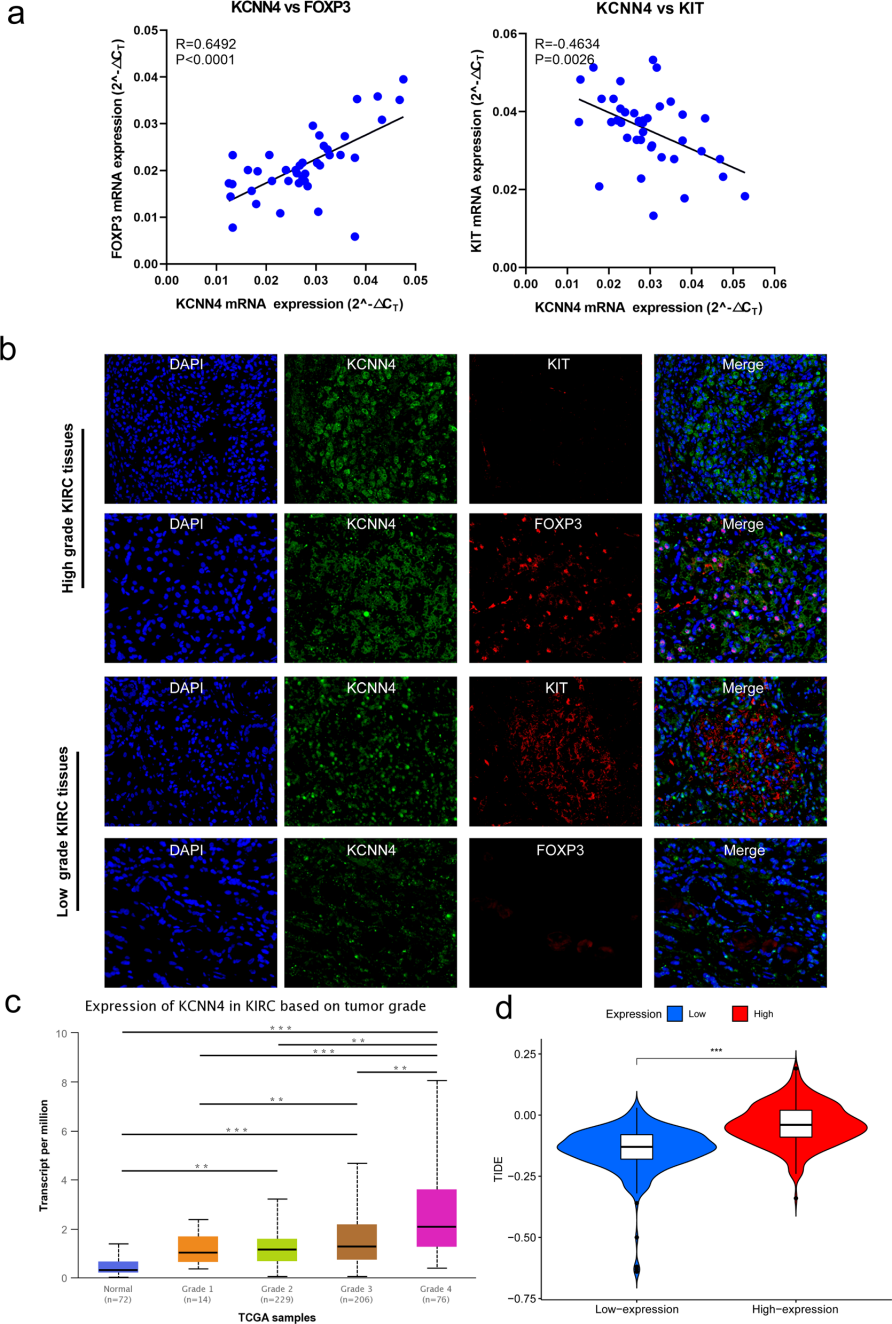
The validation of the mRNA and protein expression relationship between KCNN4 and FOXP3, KCNN4 and KIT respectively. **a** The qRT-PCR results of the mRNA expression of KCNN4, FOXP3, and KIT. **b** The immunofluorescence staining of KCNN4, FOXP3, and KIT in high and low-grade ccRCC samples. **c** The expression of KCNN4 in KIRC based on tumor grade. **d** The relationship between KCNN4 expression and TIDE score

We then used our 40 ccRCC samples to verify the correlation between KCNN4 and FOXP3, KCNN4 and KIT (Fig. 7a). Consistent with bioinformatic analysis, KCNN4 had positive correlation with FOXP3 (R = 0.6413, p < 0.01) and negative correlation with KIT (R = -0.4261, p < 0.01). Besides, we performed immunofluorescence assay with high grade tissues and low-grade tissues to study the expression difference (Fig. 7b). We found that the fluorescent signals of KCNN4 were stronger in high grade tissues. In addition, higher KCNN4 were accompanied by higher FOXP3 staining and lower KIT staining. On the contrary, lower KCNN4 were accompanied by higher KIT staining and lower FOXP3 staining. UALCAN database showed that high expression level of KCNN4 may be found in high tumor grade (Fig. 7c). Besides, high KCNN4 expression predicted worse efficiency of ICIs for KIRC patients. This is consistent with the above TME results. Higher expression of KCNN4 may lead to higher immunescore and stromalscore, which refers to weaker immunosurveillance and lower sensitiveness to ICIs.

Given the important role of KCNN4 in immune cells infiltration, we then explore the possible regulatory mechanisms from the ceRNA network. According to Fig. 2, we knew the potential role of C1RL-AS1 in sponging has-miR-16-5p. Hence, we adopted Starbase v3.0 [13] to predict the potential binding sites (Figure S4a). We found that both C1RL-AS1 and KCNN4 have binding sites to has-miR-16-5p, which further confirms the possibility of endogenous competition between C1RL-AS1 and KCNN4. Besides, the expression of miR-16-5p is higher than C1RL-AS1 and KCNN4 in 517 KIRC samples (Figure S4b).

Collectively, as a ceRNA hub gene, KCNN4 may function as a tumor promoter by recruiting Tregs and diminishing resting mast cells, and so inhibiting the anti-tumor immunity of the host. More mechanism study on the regulatory role of hsa-miR-16-5p to KCNN4 will bring ccRCC patients more therapeutic choices.

## Discussion

ccRCC is one of the most challenging tumors with a high recurrence rate after initial resection [14]. Molecular and cellular components play an important role in tumorigenesis and recurrence and are often considered as potential prognostic factors [15]. The aberrant expression of important genes in tumors and the difference of infiltration of immune cells between tumor and normal tissues have attracted our interest. However, previous studies on this aspect are scarce. In the present study, we found significant differences in tumor immune cell infiltration and ceRNAs expression between tumor and normal tissues. On this basis, two prediction models with high efficacy were constructed, which are helpful for assessing the prognosis of ccRCC patients. By comparing the correlation of ceRNAs with immune cells, we found the potential role of KCNN4 in regulating Tregs and resting mast cells. Furthermore, the lncRNA C1RL-AS1 may sponge hsa-miR-16-5p to release the inhibition of KCNN4. We subsequently applied multiple databases for multidimensional validation to verify the reliability of our results.

KCNN4 also known as KCa3.1 is part of a potentially heterotetrameric voltage-independent potassium channel that is activated by intracellular calcium. The role of the voltage-gated K^+^ (Kv) channels and membrane potential in the activation of T lymphocytes was established more than three decades ago [16]. As a Ca^2+^-activated K^+^ channel, KCNN4 presents in much lower numbers in resting naive T cells, but at substantially elevated numbers in activated T cells [17]. Besides, activation of K^+^ channels, accompanied with subsequent hyperpolarization of the cell membrane, enhances mast cell degranulation. Loss of KCNN4 expression significantly impairs mast cell volume regulation [18]. In our study, we found that KCNN4 showed positive and negative correlations with the infiltration of Tregs and resting mast cells, respectively. We hypothesize that elevated KCNN4 expression implies an over-activation of potassium channels. This, in turn, can lead to the activation of T cells with mast cells. Thus, Treg cell infiltration is elevated, while resting mast cell infiltration is reduced due to being activated.

ccRCC has been proven to be a highly immune-infiltrated tumor in multiple genomic and clinical studies [19, 20], and ccRCC is the most highly T cell infiltrated tumor type when compared with 18 other malignancies [21]. The functions of Tregs and mast cells in immune therapeutic response to ccRCC have been highlighted recently. There is a consensus that Tregs are associated with negative outcomes [21]. Besides, a meta-analysis suggested that high Foxp3 (+) Tregs infiltration was significantly associated with shorter OS and more advanced tumor stage in ccRCC [22]. In addition to Tregs, the recruitment of mast cells has been demonstrated to result in increased RCC angiogenesis in both in vitro and in vivo studies. Mechanically, RCC recruited mast cells by modulating PI3K →  AKT →  GSK3β →  AM signaling [23].

In addition to the regulation of immune cells, studies on the function of KCNN4 in malignant tumors have been continuously reported in recent years. KCNN4 promotes papillary thyroid cancer cells progression via inducing epithelial-mesenchymal transition and restraining apoptosis, which suggests that KCNN4 may be a reliable diagnostic and prognostic biomarker for PTC patients [24]. In pancreatic ductal adenocarcinoma, KCNN4 was reported to interact with GABRP to induce Ca2+ entry, which leads to the activation of NF-κB signaling and ultimately promotes macrophage infiltration [25]. As early as 2015, a study found that KCNN4 expression was upregulated in ccRCC and that high expression was associated with a high risk of metastasis and poor prognosis [26].

In this study, we adopted hypergeometric testing and correlation analysis to construct the ceRNA networks. In which, KCNN4 and C1RL-AS1 were found to be significantly correlated with hsa-miR-16-5p. The further results exposed the potential binding sites between miR-16-5p and KCNN4, miR-16-5p and C1RL-AS1, respectively. miR-16-5p was reported to be sponged by SNHG16, which served as a ceRNA, and so induce the derepression of its target gene SMAD5 and result in potentiation of the TGF-β1/SMAD5 pathway to upregulate the expression of CD73 in Vδ1 T cells [27]. C1RL-AS1 probably promoted the malignant phenotype of gastric cancer via the AKT/β-catenin pathway by downregulating c-Myc [28].

We should acknowledge that our study inevitably has some limitations. First, we did not consider the heterogeneity of the immune microenvironment associated with the location of immune infiltration. Heterogeneity of histological subtypes could influence the accuracy and generalization of prediction models. Second, all data series downloaded for predictive model construction were from Western countries. Therefore, caution should be exercised when applying the findings of this study to patients in Asian countries. Besides, we just utilized the TIDE dataset to estimate the ICIs efficiency and we should enroll eligible patients treated with ICIs drugs to compare the indicative value of risk score. Last but not least, this study is only a multidimensional correlation study, not a biological mechanism study. However, despite its limitations, the present study is the first establishment of prognostic model to predict the survival of ccRCC patients based on ccRCC-specific tumor-infiltrating immune cells and ceRNA networks. In the future, more studies should be conducted to improve the model and supply more therapeutic choices which contribute to the efficiency of immune response.

## Conclusions

In this study, two models were constructed to predict the survival and prognosis of ccRCC patients based on tumor-infiltrating immune cells and ceRNA networks, and their utility was demonstrated by their significant AUC values. The proposed prediction nomograms may provide more comprehensive and effective clinical information for improving the personalized management of ccRCC patients. In addition, this study hypothesized that KCNN4 could recruit Tregs and decrease resting mast cells, which could lead to tumor progression as well as poor prognosis. More regulatory studies on this ceRNA network for C1RL-AS1/has-miR-16-5p/KCNN4 will provide more immune adjuvant treatment options for patients with advanced ccRCC.

## Supplementary Information

**Additional file 1: Figure S1.** The heatmap and the volcano plot of differentially expressed lncRNA, miRNA and mRNA between 539 tumor and 72 normal samples.

**Additional file 2: Figure S2.** Kaplan–Meier survival curves of ceRNA hub genes.

**Additional file 3: Figure S3.** The expression pattern of immune cells in ccRCC. The composition (**a**), heatmap (**b**), and violin plot (**c**) of immune cells estimated by CIBERSORT algorithm in ccRCC. CIBERSORT: Cell type identification by estimating relative subsets of RNA transcripts.

**Additional file 4: Figure S4.** The potential competitive endogenous regulating relationship between KCNN4, has-miR-16-5p, and C1RL-AS1 estimated by the Starbase database. **a** The potential binding sites between C1RL-AS1 and has-miR-16-5p, KCNN4 and has-miR-16-5p. **b** The gene expression level of these three ceRNAs.

## Data Availability

The original contributions presented in the study are included in the article/Supplementary Material. Further inquiries can be directed to the corresponding authors.

## Abbreviations

KIRC: Kidney renal clear cell carcinoma
ceRNA: Competitive endogenous RNA
TCGA: The Cancer Genome Atlas database
AUC: The receiver operating characteristic curve
Tregs: T cells regulatory
ccRCC: Clear cell renal cell carcinoma
HGs: Hub genes
AIC: Akaike information criterion
ROC: Receiver operating characteristic
GSEA: Gene set enrichment analysis
GO: Gene Ontology
OS: Overall survival
ICIs: Immune checkpoint inhibitors
TME: Tumor microenvironment

## References

[1] Siegel RL, Miller KD, Jemal A (2020). Cancer statistics. CA Cancer J Clin.

[2] Msaouel P, Malouf GG, Su X, Yao H, Tripathi DN, Soeung M (2020). Comprehensive molecular characterization identifies distinct genomic and immune hallmarks of renal medullary carcinoma. Cancer Cell.

[3] Mantia CM, McDermott DF (2019). Vascular endothelial growth factor and programmed death-1 pathway inhibitors in renal cell carcinoma. Cancer.

[4] Escudier B (2019). Combination therapy as first-line treatment in metastatic renal-cell carcinoma. N Engl J Med.

[5] Waldmann TA (2018). Cytokines in cancer immunotherapy. Cold Spring Harb Perspect Biol.

[6] Wu T, Dai Y (2017). Tumor microenvironment and therapeutic response. Cancer Lett.

[7] Salmena L, Poliseno L, Tay Y, Kats L, Pandolfi PP (2011). A ceRNA hypothesis: the Rosetta Stone of a hidden RNA language?. Cell.

[8] Qu L, Ding J, Chen C, Wu ZJ, Liu B, Gao Y (2016). Exosome-transmitted lncARSR promotes sunitinib resistance in renal cancer by acting as a competing endogenous RNA. Cancer Cell.

[9] Liu Y, Li X, Zhang C, Zhang H, Huang Y (2020). LINC00973 is involved in cancer immune suppression through positive regulation of Siglec-15 in clear-cell renal cell carcinoma. Cancer Sci.

[10] Liu H, Zhu Z, Fang J, Liu T, Zhang Z, Zhao C (2020). The ceRNA network has potential prognostic value in clear cell renal cell carcinoma: a study based on TCGA database. Biomed Res Int.

[11] Zhao K, Zhang Q, Wang Y, Zhang J, Cong R, Song N (2020). The construction and analysis of competitive endogenous RNA (ceRNA) networks in metastatic renal cell carcinoma: a study based on The Cancer Genome Atlas. Transl Androl urol.

[12] Li B, Severson E, Pignon JC, Zhao H, Li T, Novak J (2016). Comprehensive analyses of tumor immunity: implications for cancer immunotherapy. Genome Biol.

[13] Li JH, Liu S, Zhou H, Qu LH, Yang JH (2014). starBase v2.0: decoding miRNA-ceRNA, miRNA-ncRNA and protein-RNA interaction networks from large-scale CLIP-Seq data. Nucleic Acids Res.

[14] Ljungberg B, Bensalah K, Canfield S, Dabestani S, Hofmann F, Hora M (2015). EAU guidelines on renal cell carcinoma: 2014 update. Eur Urol.

[15] Rodina A, Wang T, Yan P, Gomes ED, Dunphy MP, Pillarsetty N (2016). The epichaperome is an integrated chaperome network that facilitates tumour survival. Nature.

[16] DeCoursey TE, Chandy KG, Gupta S, Cahalan MD (1985). Voltage-dependent ion channels in T-lymphocytes. J Neuroimmunol.

[17] Nicolaou SA, Neumeier L, Peng Y, Devor DC, Conforti L (2007). The Ca(2+)-activated K(+) channel KCa3.1 compartmentalizes in the immunological synapse of human T lymphocytes. Am J Physiol Cell physiol.

[18] Shumilina E, Lam RS, Wölbing F, Matzner N, Zemtsova IM, Sobiesiak M (2008). Blunted IgE-mediated activation of mast cells in mice lacking the Ca2+-activated K+ channel KCa3 1. J Immunol..

[19] Yoshihara K, Shahmoradgoli M, Martínez E, Vegesna R, Kim H, Torres-Garcia W (2013). Inferring tumour purity and stromal and immune cell admixture from expression data. Nat Commun.

[20] Thompson RH, Dong H, Lohse CM, Leibovich BC, Blute ML, Cheville JC (2007). PD-1 is expressed by tumor-infiltrating immune cells and is associated with poor outcome for patients with renal cell carcinoma. Clin Cancer Res.

[21] Şenbabaoğlu Y, Gejman RS, Winer AG, Liu M, Van Allen EM, de Velasco G (2016). Tumor immune microenvironment characterization in clear cell renal cell carcinoma identifies prognostic and immunotherapeutically relevant messenger RNA signatures. Genome Biol.

[22] Shang B, Liu Y, Jiang SJ, Liu Y (2015). Prognostic value of tumor-infiltrating FoxP3+ regulatory T cells in cancers: a systematic review and meta-analysis. Sci Rep.

[23] Chen Y, Li C, Xie H, Fan Y, Yang Z, Ma J (2017). Infiltrating mast cells promote renal cell carcinoma angiogenesis by modulating PI3K→AKT→GSK3β→AM signaling. Oncogene.

[24] Wen J, Lin B, Lin L, Chen Y, Wang O (2020). KCNN4 is a diagnostic and prognostic biomarker that promotes papillary thyroid cancer progression. Aging.

[25] Jiang SH, Zhu LL, Zhang M, Li RK, Yang Q, Yan JY (2019). GABRP regulates chemokine signalling, macrophage recruitment and tumour progression in pancreatic cancer through tuning KCNN4-mediated Ca(2+) signalling in a GABA-independent manner. Gut.

[26] Rabjerg M, Oliván-Viguera A, Hansen LK, Jensen L, Sevelsted-Møller L, Walter S (2015). High expression of KCa3 1 in patients with clear cell renal carcinoma predicts high metastatic risk and poor survival. PloS ONE.

[27] Ni C, Fang QQ, Chen WZ, Jiang JX, Jiang Z, Ye J (2020). Breast cancer-derived exosomes transmit lncRNA SNHG16 to induce CD73+γδ1 Treg cells. Signal Transduct Target Ther.

[28] Zhen-Hua W, Yi-Wei G, Li-Qin Z, Jie-Yun Z, Zhe G, Wei-Jian G (2020). Silencing of LncRNA C1RL-AS1 suppresses the malignant phenotype in gastric cancer cells via the AKT/β-Catenin/c-Myc pathway. Front Oncol.

