# Supplementary figures and images for: KCNN4 may weaken anti-tumor immune response via raising Tregs and diminishing resting mast cells in clear cell renal cell carcinoma
Source: Cancer Cell Int. 2022 Jun 10;22:211. doi: 10.1186/s12935-022-02626-7 (PMC9185981; doi:10.1186/s12935-022-02626-7)

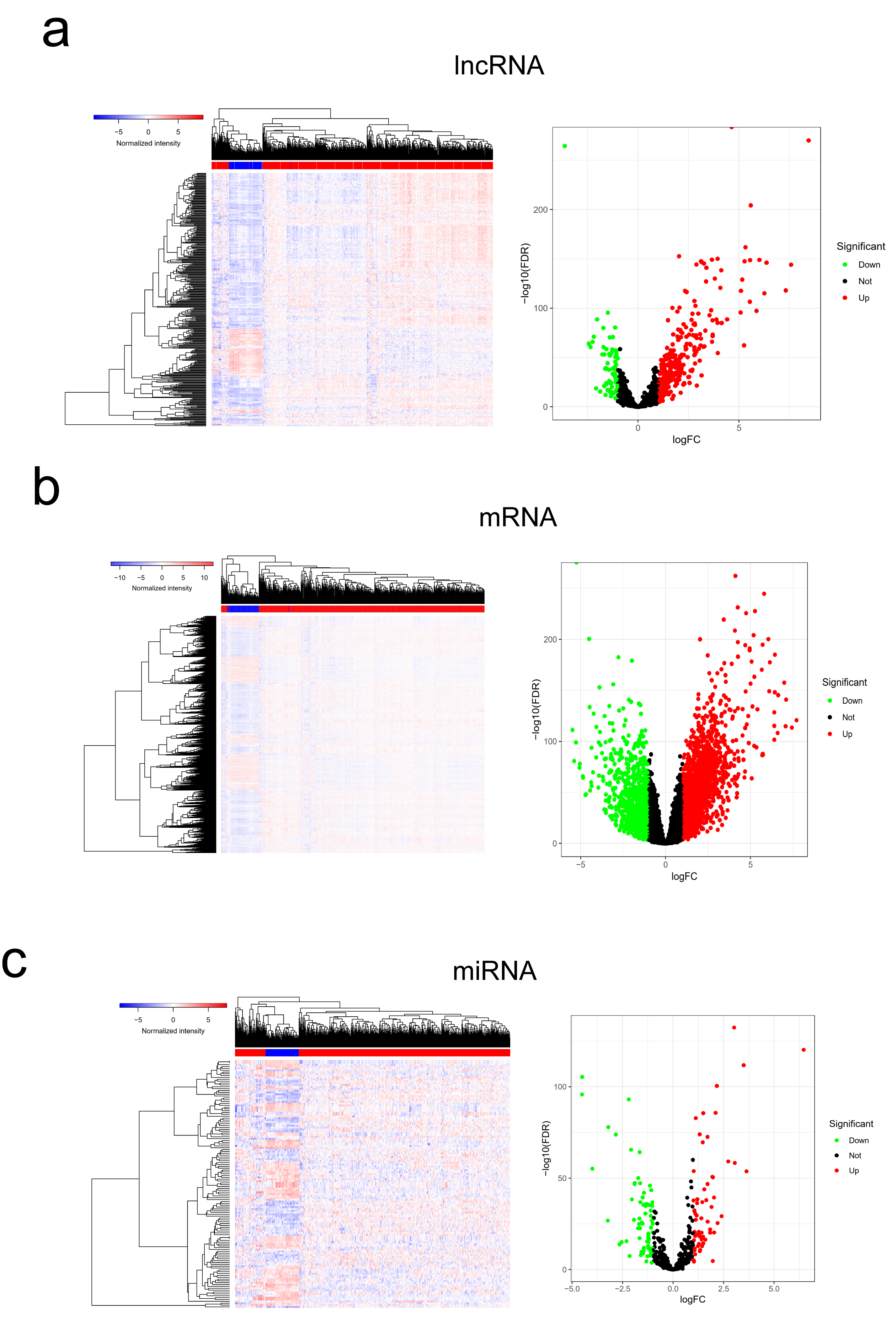

Supplement: Supplementary file 1 — Additional file 1: Figure S1. The heatmap and the volcano plot of differentially expressed lncRNA, miRNA and mRNA between 539 tumor and 72 normal samples. [file 12935_2022_2626_MOESM1_ESM.tif]

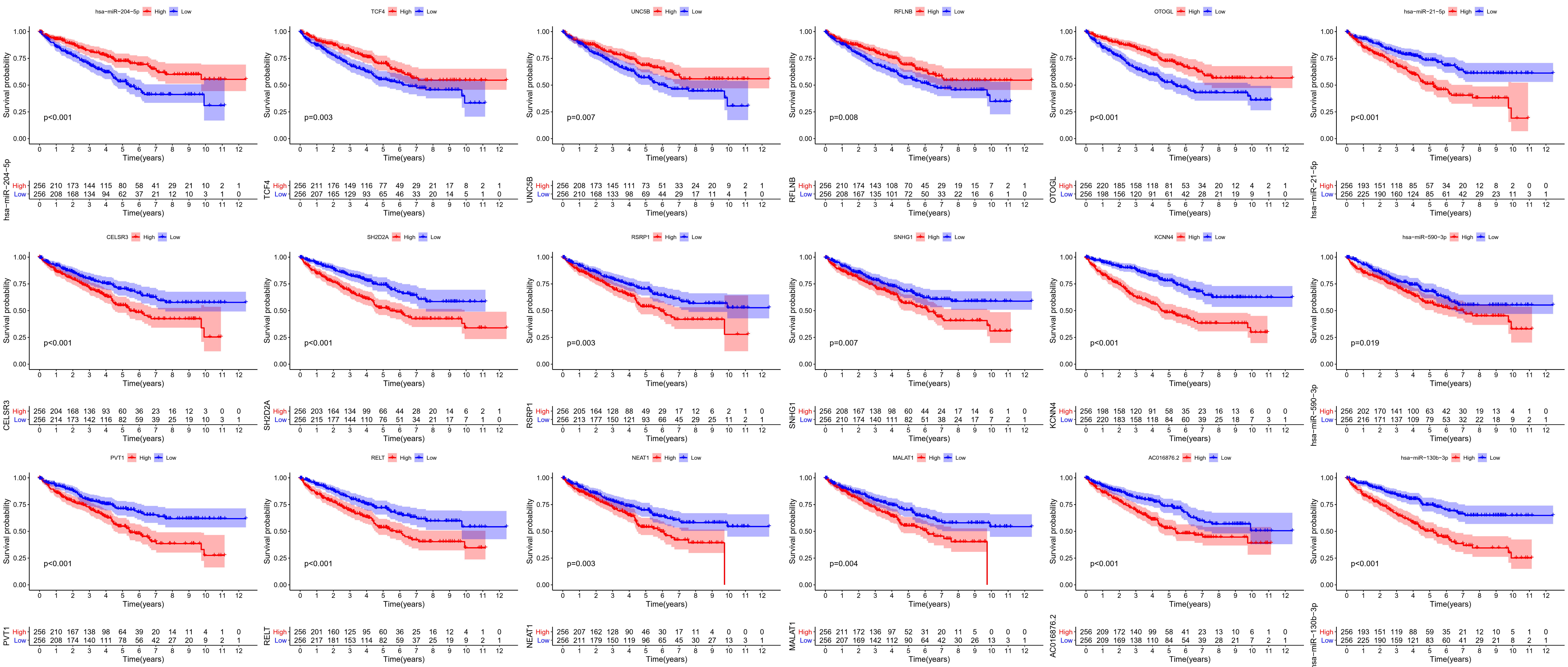

Supplement: Supplementary file 2 — Additional file 2: Figure S2. Kaplan–Meier survival curves of ceRNA hub genes. [file 12935_2022_2626_MOESM2_ESM.tif]

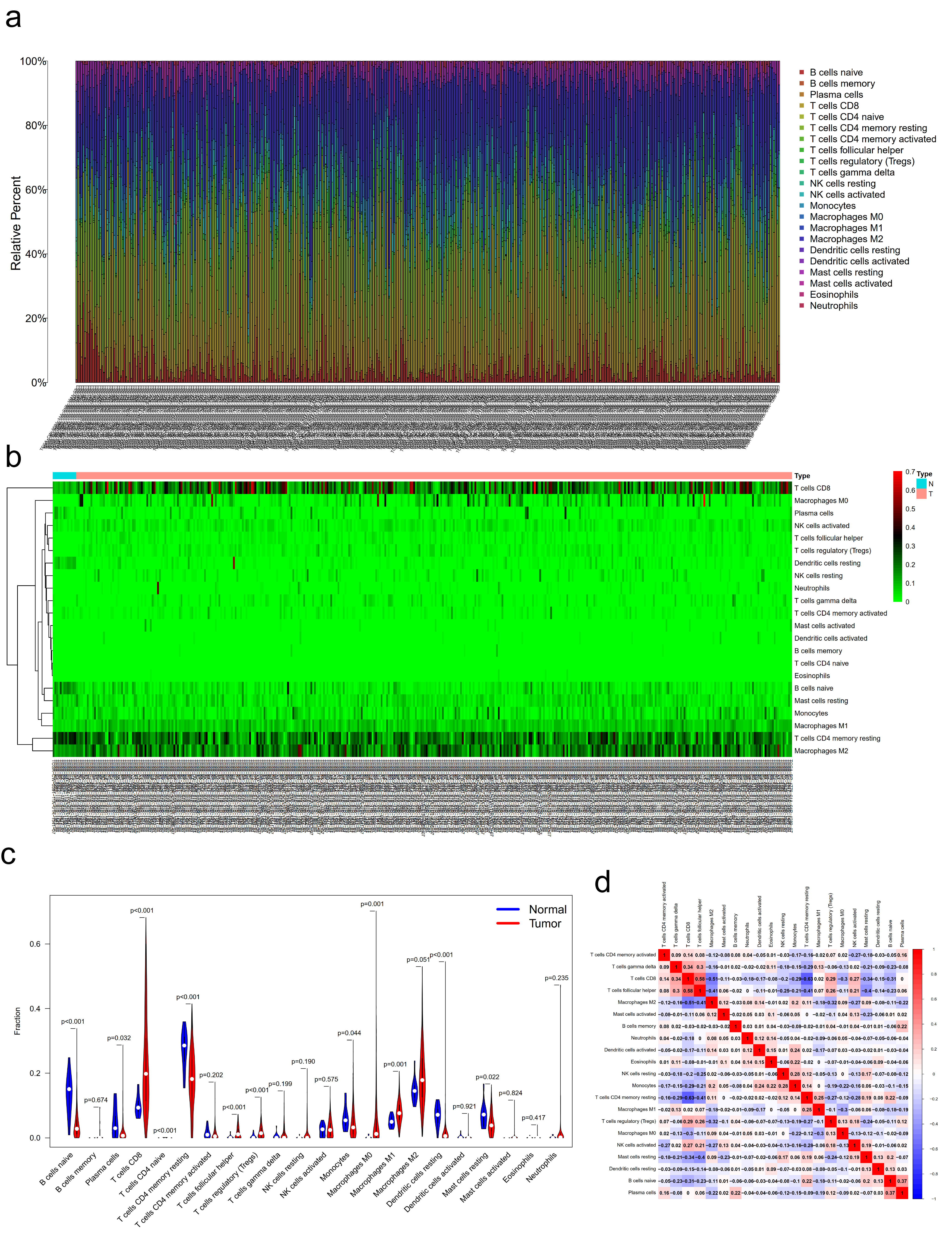

Supplement: Supplementary file 3 — Additional file 3: Figure S3. The expression pattern of immune cells in ccRCC. The composition (a), heatmap (b), and violin plot (c) of immune cells estimated by CIBERSORT algorithm in ccRCC. CIBERSORT: Cell type identification by estimating relative subsets of RNA transcripts. [file 12935_2022_2626_MOESM3_ESM.tif]

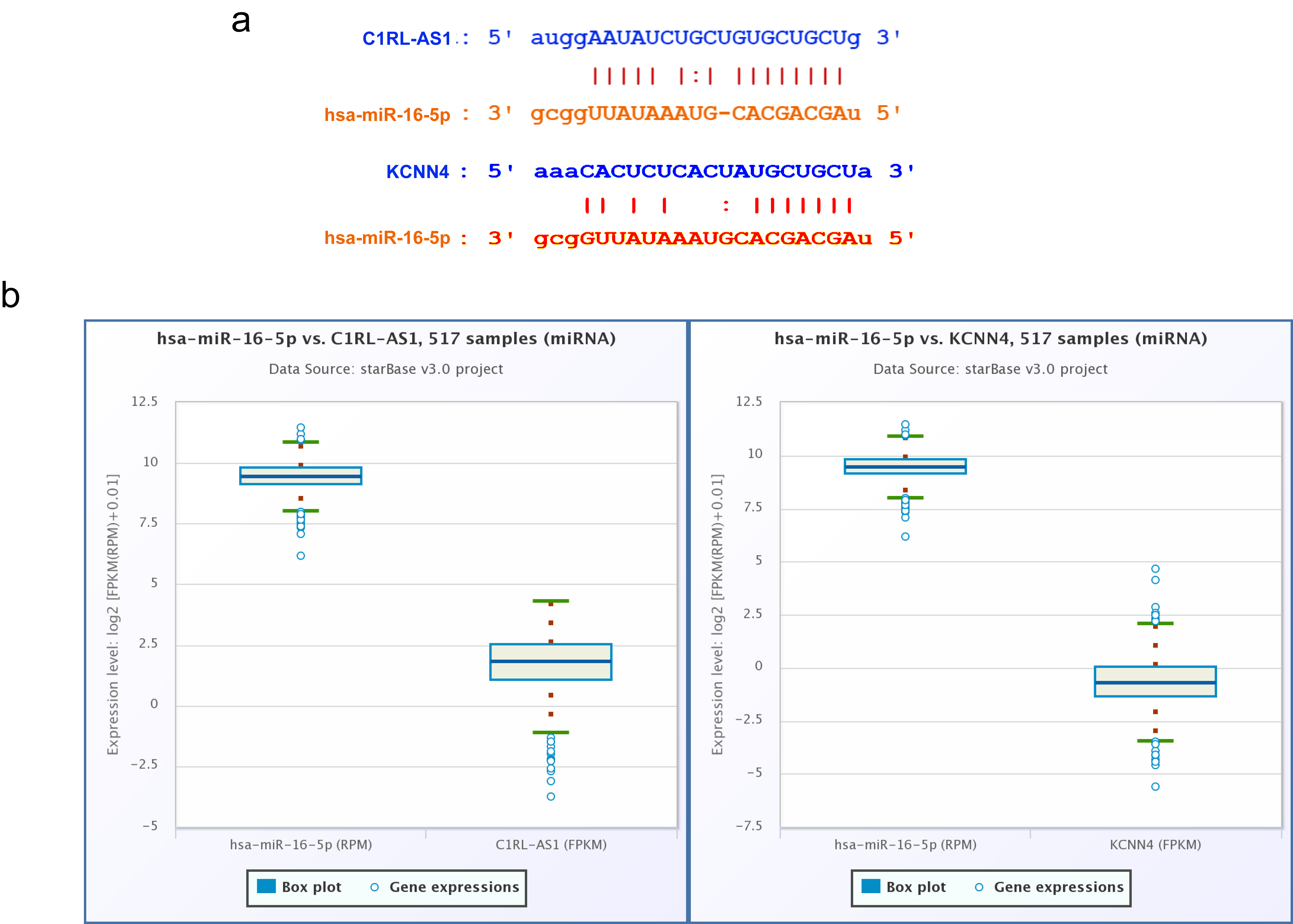

Supplement: Supplementary file 4 — Additional file 4: Figure S4. The potential competitive endogenous regulating relationship between KCNN4, has-miR-16-5p, and C1RL-AS1 estimated by the Starbase database. a The potential binding sites between C1RL-AS1 and has-miR-16-5p, KCNN4 and has-miR-16-5p. b The gene expression level of these three ceRNAs. [file 12935_2022_2626_MOESM4_ESM.tif]
